# High-Order Dielectric Metasurfaces for High-Efficiency Polarization Beam Splitters and Optical Vortex Generators

**DOI:** 10.1186/s11671-017-2279-2

**Published:** 2017-08-29

**Authors:** Zhongyi Guo, Lie Zhu, Kai Guo, Fei Shen, Zhiping Yin

**Affiliations:** 1 School of Computer and Information, Hefei University of Technology, Hefei, 230009 China; 2 Academy of Opto-Electronic Technology, Hefei University of Technology, Hefei, 230009 China

**Keywords:** Metasurface, Beam splitters, Phase modulation, Optical vortices

## Abstract

In this paper, a high-order dielectric metasurface based on silicon nanobrick array is proposed and investigated. By controlling the length and width of the nanobricks, the metasurfaces could supply two different incremental transmission phases for the X-linear-polarized (XLP) and Y-linear-polarized (YLP) light with extremely high efficiency over 88%. Based on the designed metasurface, two polarization beam splitters working in high-order diffraction modes have been designed successfully, which demonstrated a high transmitted efficiency. In addition, we have also designed two vortex-beam generators working in high-order diffraction modes to create vortex beams with the topological charges of 2 and 3. The employment of dielectric metasurfaces operating in high-order diffraction modes could pave the way for a variety of new ultra-efficient optical devices.

## Background

In recent years, the full control of electromagnetic waves has been an emerging area of research. For the quest to realize such control, metamaterials have attracted significant attentions for their novel physical properties, which could be artificially engineered as desires by structuring their constituents [1]. So far, metamaterials have been used to achieve many excellent optical properties, such as negative refraction, zero-refraction, and slow-light. However, three-dimensional metamaterial has many drawbacks, such as high intrinsic losses and fabrication difficulty, which restrict its real applications. With the developments of nanotechnology, two-dimensional metamaterials, or so-called metasurfaces, have been proposed to avoid these drawbacks due to their ultrathin subwavelength structures, relatively easy fabrication and conformal integrations with systems [2, 3]. Metasurfaces typically consist of an array of optical resonators with subwavelength period and function as interface discontinuities. It could introduce an abrupt change in the amplitude or phase of the impinging beam by designing the geometry of the resonator. Based on this concept, various metasurfaces with different functions have been implemented, including tunable waveguide [4, 5], wave-plates [6, 7], lens [8–11], anomalous refraction [12, 13], compact vortex generators [14–16], and high-resolution holograms [17–19].

Although metasurface exhibits much better efficiency compared with three-dimensional metamaterials, the loss should still be considered seriously due to the common use of metal. Hence, there are some improved methods to increase the transmission efficiency, including the Huygens’ metasurfaces and all-dielectric metasurfaces. The Huygens’ metasurfaces could avoid low efficiency; nevertheless, the fabrication of the three-dimensional structures still hinders it applications in reality [20]. Fortunately, dielectric metasurfaces could be optimized to simultaneously possess overlapping electric and magnetic resonances at the same frequencies and thus enable full 2*π* phase control with high transmission efficiency [21–27]. However, most of the demonstrated optical devices in the previous works use the ±1*st* order diffraction modes to manipulate the wavefront of light rather than the high order modes [28–30]. Recently, a novel approach has been proposed to control the incident wavefront and operates in high order modes by modulating the discrete phase; still, they obtained quite low transmission efficiencies due to the intrinsic Ohmic loss of metal [31, 32].

In this work, we propose a dielectric metasurface to manipulate the wavefront operating in high-order diffraction modes with extremely high transmission efficiency. Based on the proposed dielectric metasurface, two polarizing beam splitters with abrupt phase discontinuities have been designed in the telecommunication band and operating in high-order modes. The polarizing beam splitters are capable of generating two different wavefronts for two orthogonal input polarizations with extremely high efficiency up to 88%. In addition, we have also designed two vortex beam generators with the topological charges of 2 and 3 to further demonstrate the capability of the designed metasurface to manipulate light in high-order diffraction modes.

## Methods 

**Fig. 1:**
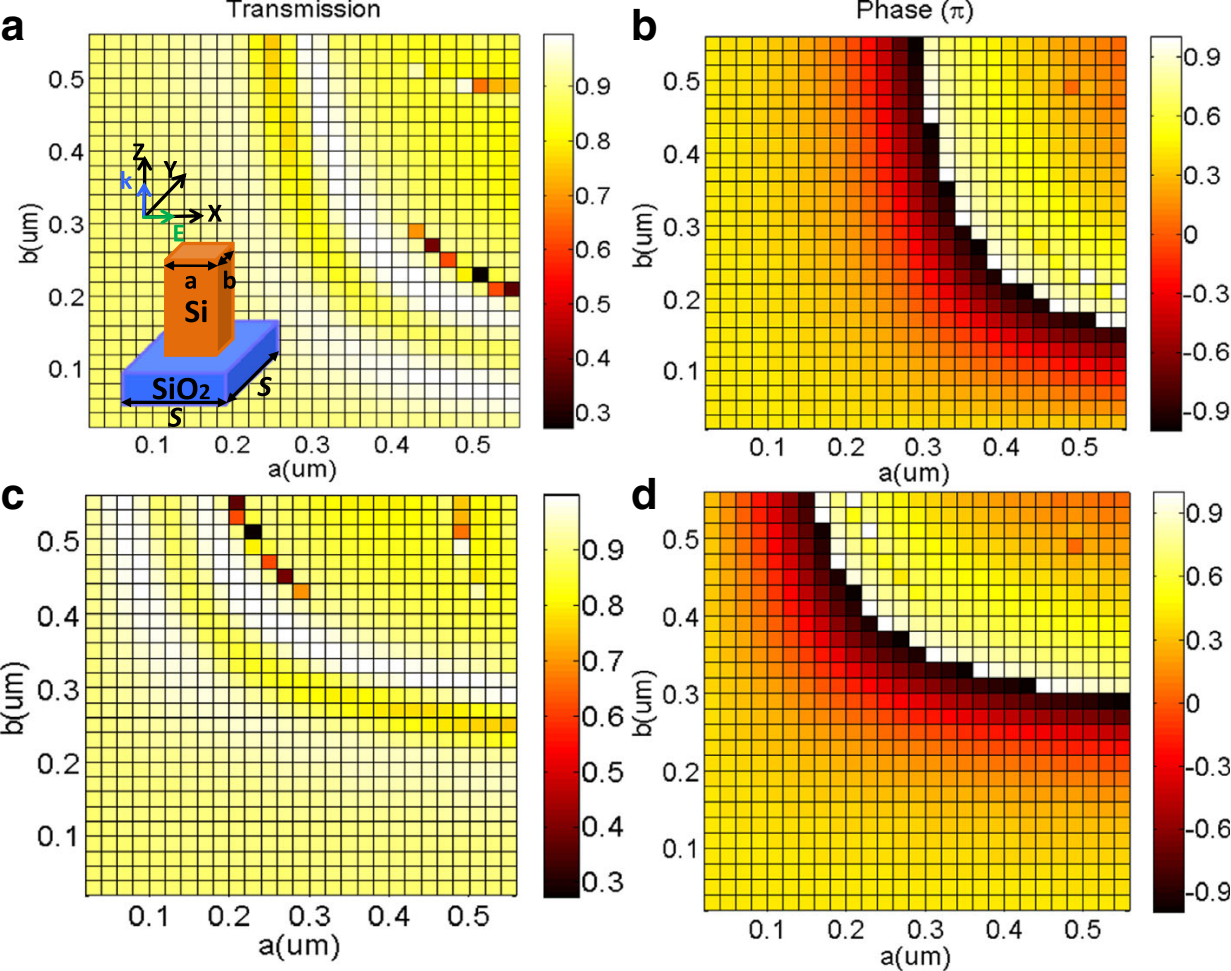
**a** The transmission efficiency and **b** the corresponding phase variations of XLP light as a function of parameters *a* and *b*. **c** The co-polarized transmission efficiency and **d** corresponding phase variations of YLP light as a function of parameters *a* and *b*. The inset in **a** schematically shows the unit cell of periodic dielectric metasurface consisting of an array of silicon nanobricks on top of SiO_2_ substrate. The thickness of silicon nanobricks and SiO_2_ substrate is set as 900 nm and 200 nm, respectively

The schematic of the designed dielectric metasurfaces is shown in the inset of Fig. 1a. It is composed of 900-nm thick crystalline silicon nanobrick etched on a 200-nm thick glass substrate, whose refractive indices are 3.48 and 1.48, respectively. Due to the high refractive index, silicon exhibits high-quality resonant properties and low intrinsic ohmic losses. Furthermore, the nanostructured silicon can be easily obtained by mature technology of semiconductor with low manufacture cost, such as EBL and FIB. The *SiO*_2_ substrate was used due to that the reflection loss and the absorption loss can be nearly neglected in the wavelength of 1500 nm. The lattice constant is chosen as *S* = 650 nm. Thus, the geometric phase of the transmitted light induced by a silicon nanorod depends on the nanobrick dimensions along X- and Y-directions. The numerical simulation is performed by FDTD (finite-different time-domain) method. In the simulations, the perfectly matched layer (PML) was added to the layer above and below a cell to function as absorbing boundary conditions. In addition, the periodic boundary conditions (PBC) have also been applied around a cell or a unit cell. The operation wavelength is chosen to be 1500 nm for the wavelength of optical communications.

By using the numerical simulation, as depicted in Fig. 1, the co-polarized transmitted efficiency and the corresponding phase variations for both X-linear-polarized (XLP) light and Y-linear-polarized (YLP) light are calculated as functions of the geometries of the silicon bricks. When the XLP light is incident to the proposed dielectric metasurface, there is high transmittance for almost all of the nanobrick dimensions, as presented in Fig. 1a. Meanwhile, Fig. 1b implies a full range of phase from 0 to 2*π* in transmission of XLP light, which could provide a full coverage of wavefront phase. More importantly, for the vast majority of dimensions, the nanobricks have over 88% co-polarized power transmission efficiency, which could be attributed to the low reflection and nearly no absorption of the dielectric metasurface at the telecommunication wavelength. The co-polarized transmission efficiency and corresponding phase variations under the YLP incidence are plotted in Fig. 1c, d, respectively. Because of the symmetry, the dependence of optical properties of dielectric metasurface on geometric dimensions for YLP light is similar with that for XLP light, which is clearly shown in Fig. 1. Hence, for YLP light, the co-polarized transmission efficiency is also higher than 88% and modulating phase range could vary from 0 to 2*π*.

In brief, a complete range of phase control from 0 to 2*π* could be effectively achieved in the case of XLP and YLP incidences by only changing the geometric dimension of nanobrick along X-direction (i.e., *a*) and Y-direction (i.e., *b*), respectively. Consequently, the range of phase control could be extended to high-order diffraction modes (i.e., from 0 to N × 2*π*) due to the periodicity of phase. To demonstrate the versatility and precise phase control of the designed nanobricks, two transmission-type optical devices with high efficiency have been proposed by well designing the metasurface with simply arrangement, including two polarizing beam splitters and an optical vortex generator.

## Results and discussion

### Designing the Polarizing Beam Splitters

On-chip polarization control is an important issue for photonic integrated circuits. The polarizing beam splitter is one of the essential optical devices used to control the polarization on a chip, which can be used to separate the input light into two orthogonal polarization components [33, 34]. According to the simulation results above, beam splitters with steerable birefringence based on the proposed dielectric metasurface could be realized, which indicates that two different phases of XLP refraction light (*φ*_*x*_) and YLP refraction light (*φ*_*y*_) could be simultaneously obtained by appropriately selecting the nanobrick diameters *a* and *b*, respectively. Thus, we here design metasurfaces and employ this novel property to realize polarizing beam splitters to distinguish two orthogonal polarizations of input light to two directions with highly transmitted efficiency up to 88%. Furthermore, the designed metasurface could work in not only the first-order but also the higher-order diffraction modes.

**Fig. 2:**
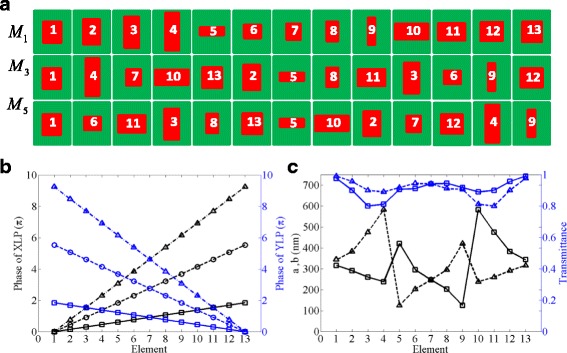
Design of the dielectric metasurfaces with three different order diffraction modes. **a** Schematics of lateral dimensions of the 13 designed nanobricks. First *line* *M*_1_: a supercell with transmitted phase ranging from 0 to 2*π*. Second line *M*_3_: a rearranged super cell with phase ranging from 0 to 3 × 2*π*. Third line *M*_5_: a rearranged super cell with phase ranging from 0 to 5 × 2*π*. **b** The simulated transmission phases of the 13 designed nanobricks of three different modes under XLP (*black lines*) and YLP (*blue lines*) incidences, respectively. **c** *a* (*black solid lines*) and *b* (*black dotted lines*) of the 13 nanobricks used in the designed metasurfaces *M*_1_. The *blue lines* represent the transmitted efficiencies of the 13 nanobricks in *M*_1_ under XLP (*solid lines*) and YLP (*dotted lines*) incidences, respectively

We design the polarizing beam splitters by 13 dielectric nanobricks with three different permutations to generate different order diffraction modes with high efficiency. In the design of metasurface 1 (*M*_1_), we discretize the phase range from 0 to 2*π* and from 2*π* to 0 into 13 nanobricks with equal step of 2*π*/13 and −2*π*/13 for X- and Y-polarized transmitted light, respectively. The lateral dimensions of the 13 selected silicon nanobricks are numbered in ascending order, as shown in the first line of Fig. 2a. Apparently, the range of phase control could be extended to high-order diffraction mode by appropriately selecting the unit cells in *M*_1_ and rearranging them. For example, if we extend the diffraction mode to the Nth order, the range of phase should cover from 0 to *N* × 2*π* and from *N* × 2*π* to 0 with a phase difference of *N* × 2*π*/13 and −*N* × 2*π*/13 between two neighboring nanobricks for X- and Y-polarized transmitted light, respectively. Therefore, the second line of Fig. 2a presents the rearranged super cells for the third order diffraction mode (*M*_3_), whose range of phase control is from 0 to 3 × 2*π* and from 3 × 2*π* to 0 with a phase difference of 3 × 2*π*/13 and −3 × 2*π*/13 between two neighboring nanobricks for X- and Y-polarized transmitted light, respectively. In addition, the metasurface (*M*_5_) for the fifth order diffraction mode is also constructed by a set of 13 dielectric nanobricks, which are also rearranged to cover entire range of phase control from 0 to 5 × 2*π* and from 5 × 2*π* to 0 with a phase difference of 5 × 2*π*/13 and −5 × 2*π*/13 between two neighboring nanobricks for X- and Y-polarized transmitted light, respectively, as presented in the third line of Fig. 2a. In order to show the idea clearly, the transmission phases of the 13 antennas in three concrete permutations under XLP and YLP light are plotted in Fig. 2b.

In addition, the transmissions of the 13 designed nanobricks under XLP and YLP light have been simulated and agree well with the theoretical prediction. Fig. 2c shows the geometrical dimensions of the silicon nanobricks and the transmitted efficiencies of the 13 nanobricks in metasurface *M*_1_ under XLP and YLP light. The co-polarized transmissions of most dielectric nanobricks are comparable and remain over 88% though there are two nanobricks’ transmissions keeping nearly 80%. These simulation results verify that our designed metasurfaces could be applied to fabricate numerous optical devices with high efficiency.

**Fig. 3:**
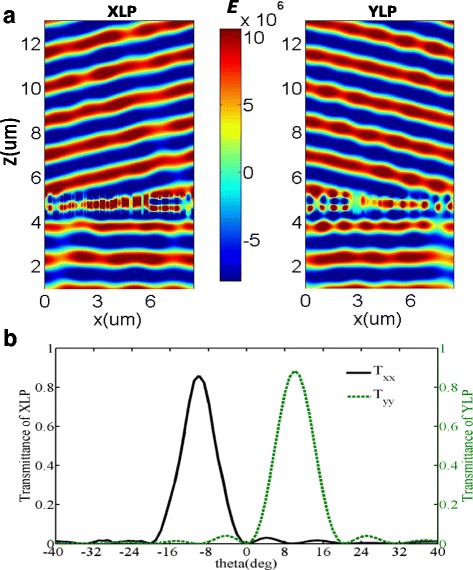
**a** The electric field distributions (*E*) of extracted transmitted XLP (*left*) and YLP (*right*) light, when a normal incident light with 45^°^ linear-polarization at the wavelength of 1500 nm transmitted through the designed metasurfaces. **b** The co-polarized transmitted efficiencies of the designed metasurfaces as the function of the transmitted angle under the illuminations of an X-polarized and Y-polarized lights, respectively

Numerical simulations of polarizing beam splitter are performed by illuminating the designed metasurfaces *M*_1_ at normal incidence with the polarized angle of 45^°^. The concrete XLP and YLP light could be extracted from the whole transmitted fields, as plotted in Fig. 3a. It is clear that there exists a well-defined wavefront and the co-polarized transmitted efficiencies of *M*_1_ are plotted as functions of transmitted angle in Fig. 3b. The peak co-polarized transmitted angles are −10.2^°^ and 10.2^°^ for transmitted XLP and YLP lights, respectively. The efficiencies of the first order are *T*_*xx*_ = 85.9% and *T*_*yy*_ = 88.4% for the transmitted XLP and YLP lights, respectively, where *T*_*xx*_ is the simulated transmission coefficient of XLP light with the XLP incidence and *T*_*yy*_ is the simulated transmission coefficient of YLP light with the YLP incidence. Compared to the transmitted efficiency of the spatially homogeneous nanobrick arrays, the conversion efficiency is slightly reduced owing to the coupling between resonators with different dimensions [35]. On the basis of the generalized Snell’s law, the diffraction angle of incident light at a gradient metasurface can be calculated by *θ*_*t*_ = sin^−1^[(*λ*_0_/*n*_*t*_ *L*) + *n*_*i*_ sin(*θ*_*i*_)/*n*_*t*_], where *n*_*t*_ and *n*_*i*_ are the refractive indexes of the media in the transmission and incident sides of the interface, respectively, *θ*_*i*_ is the incident angle, *λ*_0_ is the wavelength of light in vacuum, and *L* is the length of a supercell [36]. Thus, the theoretical results of the first order diffraction angles are ±10.22^°^. Numerical simulation and theory agree well with each other. That is to say the designed device can serve as a polarizing beam splitter with a proper successive treatment. Furthermore, the incident wavefront has almost not been affected by the reflection light from the metasurface, which verifies that all of the incident light could be transmitted from the metasurfaces with extremely high efficiency.

**Fig. 4:**
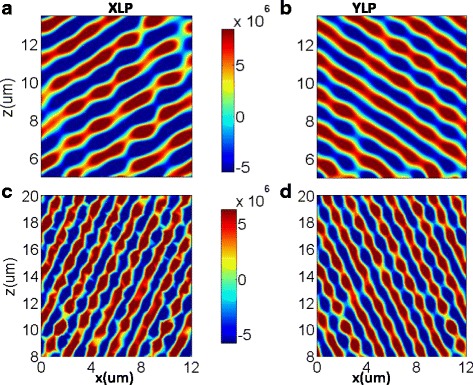
The electric field distributions of extracted transmitted XLP (*left*) and YLP (*right*) under the normal incidence of 45^°^ linear-polarization light to the metasurfaces of *M*_3_(**a**, **b**) and *M*_5_(**c**, **d**), respectively

**Fig. 5:**
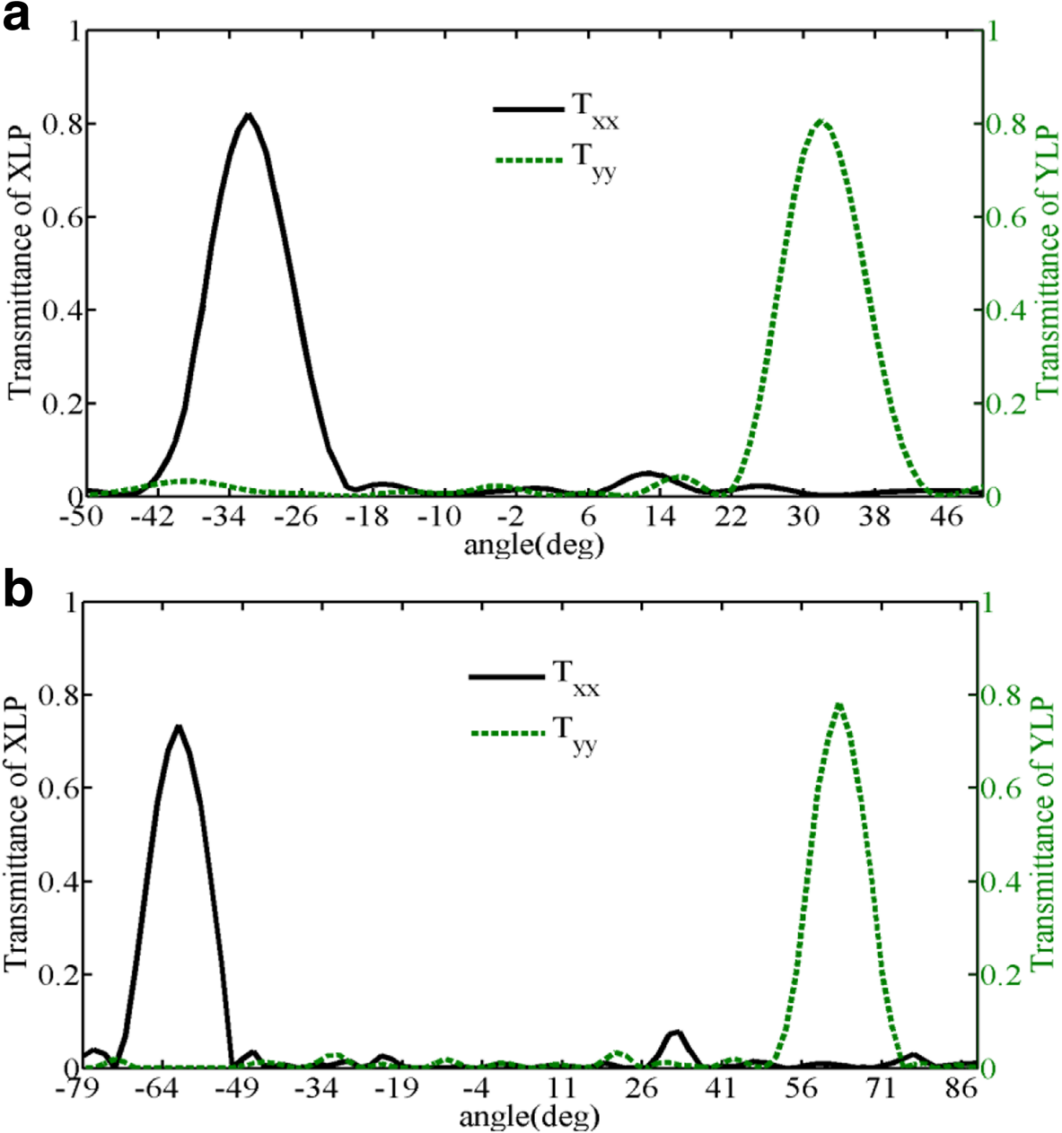
The co-polarized transmitted efficiencies of the designed metasurfaces composed of rearranged supercell **a** *M*_3_ and **b** *M*_5_ as functions of the transmitted angle under the illumination of a X-polarized and Y-polarized light, respectively

For comparison, Fig. 4 shows the concrete XLP and YLP transmitted electric field distributions of the other two rearranged dielectric metasurfaces made of new designed supercells (*M*_3_ and *M*_5_) under the 45^°^ linear-polarized incident light. Since the transmitted phase range of the two supercells has been changed, the diffraction angles of *M*_3_ and *M*_5_ are theoretically calculated to be ±32.18^°^ and ±62.56^°^, respectively. In Fig. 4a, b, there exist two well-defined phase fronts with the third order diffraction angles of −32^°^ and 32^°^ for transmitted XLP and YLP lights, respectively. In Fig. 4c, d, the fifth order diffraction angle is −63^°^ and 63^°^ for transmitted XLP and YLP lights, respectively. Furthermore, the simulated co-polarized transmitted efficiencies of the designed metasurfaces composed of rearranged supercell *M*_3_ and *M*_5_ have also been illustrated in Fig. 5a, b, respectively. The peak transmitting angles match well with the theoretical diffraction angles calculated by the generalized Snell’s law, and the co-polarized diffraction efficiencies of the third order are 82 and 84% for transmitted XLP and YLP lights. However, the co-polarized diffraction efficiencies of the fifth order are just 73.5 and 78.4% for transmitted XLP and YLP lights, which is essentially caused by the undesired EM coupling between neighboring nanobricks with different geometries. Therefore, the designed metasurfaces could work well in higher-order diffraction modes by just modifying the arrangement of the 13 dielectric nanobricks. More importantly, it is demonstrated that the diffraction mode could be customized by controlling the phase difference between adjacent dielectric nanobricks in a supercell.

### Designing the Optical Vortex Generators

**Fig. 6:**
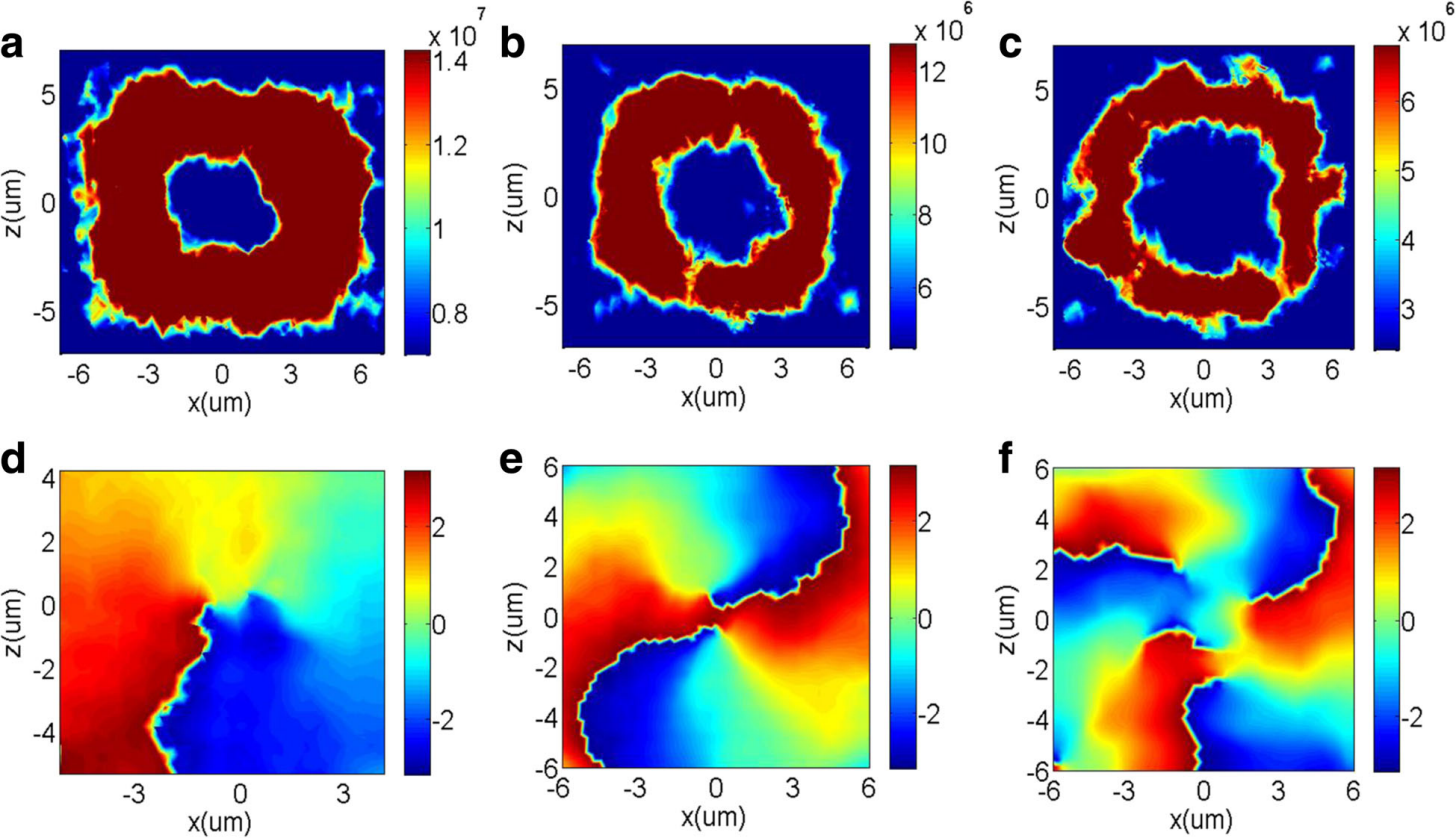
**a**–**c** The transmitted intensity distributions and **d**–**f** the phase wavefronts of the generated vortex beams at *z* = 10*μm*with topological charge of *l* = 1 , 2 , 3 based on the metasurfaces *M*_1_, *M*_2_, and *M*_3_ under the X-polarized incidence, respectively

The optical vortex beam has a helical wavefront and carries an orbital angular momentum of *lℏ* [37, 38], which make it show great promises in high-resolution lithography [39, 40], optical trapping [41, 42], optical communication [43, 44], and so on. Here, the topological charge *l* is the number of twists of the wavefront and *ℏ* is the reduced Planck constant. The vortex beam with the topological charge of 1 can be generated by metasurfaces with spiral phase profile ranging from 0 to 2*π* with identical phase increment along the azimuthal direction. Therefore, to further demonstrate the capability of the designed metasurface to manipulate the transmitted phase and diffraction mode, we design a vortex generator that can convert an incident homogeneous Gaussian beam into a vortex beam. To achieve this goal, we arrange the 13 dielectric nanobricks of *M*_1_ into the 13 sectors to introduce a gradient phase increment of 2*π*/13across the azimuthal direction. The transmitted intensity profiles under XLP incidence at *z* = 10 *μm* are shown in Fig. 6a and have the characteristic intensity minimum at the center corresponding to a phase singularity. The spatial phase patterns with an evident abrupt phase jump from −*π* to *π* within a 2*π* azimuthal range are shown in Fig. 6d, which indicates that the topological charge of the optical devices in Fig. 6d is 1.

In addition, we design other two vortex generators to generate vortex beams by changing the arrangement of the nanobricks in *M*_1_. These two vortex beam generators possess the topological charges of 2 and 3, respectively. Their transmitted intensity profiles under XLP incidence are shown in Fig. 6b, c, respectively. The concrete design approaches are modulating the phase difference of the nanobricks to be 4*π*/13 and 6*π*/13between two neighboring dielectric nanobricks, which are defined as *M*_2_ and *M*_3_. Therefore, the instantaneous spatial phase profiles in Fig. 6e, f possess two and three evident abrupt phase jumps from −*π* to *π*, respectively. Switching the incident polarization from XLP to YLP does not change the output intensity pattern, but the twisting direction of the helical wavefront will be reverse due to the diminishing phase difference between the neighboring nanobricks. Furthermore, it should be noted that the higher-order phase profiles could also be generated by our designed dielectric metasurfaces.

## Conclusions

In conclusion, we have demonstrated dielectric gradient metasurfaces consist of periodic arrangement of differently sized silicon nanobricks, which could transmit the input light with full range of manipulating phase from 0 to 2*π* and extremely high efficiency (over 88%) at telecommunication wavelength. Based on the designed dielectric metasurfaces, novel polarizing beam splitters working in the higher order diffraction modes are proposed to separate two orthogonal input polarized lights to arbitrary different directions. In addition, we have also designed two vortex beam generators working in the higher-order diffraction modes with different topological charges. Our work could also easily be extended to the design of other optical transmitting devices with high efficiency.
